# Low Incidence and Mortality by SARS-CoV-2 Infection Among Healthcare Workers in a Health National Center in Mexico: Successful Establishment of an Occupational Medicine Program

**DOI:** 10.3389/fpubh.2021.651144

**Published:** 2021-04-13

**Authors:** Miguel Ángel Salazar, Leslie Chavez-Galan, Armando Castorena-Maldonado, Maribel Mateo-Alonso, Nadia Otilia Diaz-Vazquez, Ana María Vega-Martínez, José Arturo Martínez-Orozco, Eduardo Becerril-Vargas, Fernando Manuel Sosa-Gómez, Hilda Patiño-Gallegos, Delfino Alonso-Martínez, Enrique López-Segundo, Fernando Vidal, Luis Joan Velasco-González, Silvia Pérez-Pulido, Patricio Santillán-Doherty, Justino Regalado-Pineda, Jorge Salas-Hernández, Ivette Buendía-Roldán

**Affiliations:** Instituto Nacional de Enfermedades Respiratorias Ismael Cosio Villegas, Mexico, Mexico

**Keywords:** SARS-CoV-2 infection, mitigate risk, healthcare workers, occupational medicine, personal protective equipment

## Abstract

**Background:** Coronavirus disease 2019 (COVID-19) is caused by severe acute respiratory syndrome coronavirus 2 (SARS-CoV-2). Healthcare workers (HCWs) constitute a population which is significantly affected by SARS-CoV-2 infection worldwide. In Mexico, the *Instituto Nacional de Enfermedades Respiratorias* (INER) is the principal national reference of respiratory diseases.

**Aim:** To evaluate the efficiency of the INER-POL-TRAB-COVID19 program to mitigate the SARS-CoV-2 infection risk among the INER-healthcare workers (INER-HCW).

**Methods:** Currently, the INER has 250 beds and 200 respiratory ventilators to support COVID-19 patients in critical condition. On March 1st, 2020, the INER-POL-TRAB-COVID19 program was launched to mitigate the SARS-CoV-2 infection risk among the INER-HCW.

**Findings:** From March 1st to October 1st, 2020, 71.5% of INER-HCWs were tested for SARS-CoV-2 infection, and 77% of them were frontline workers. Among the tested INER-HCWs, 10.4% were positive for SARS-CoV-2 infection. Nonetheless, nosocomial infection represented only 3.8% of the cases and the mortality was null. Fifty-three of INER-HCWs positive to SARS-CoV-2 had a negative test 42–56 days post-diagnosis and were returned to service. Finally, although a change in the PPE implemented on May 11th, 2020, the incidence of SARS-CoV-2 infection was not affected.

**Conclusion:** INER has a lower incidence of HCWs infected with SARS-CoV-2 as compared to the mean of the national report. The implementation of the INER-POL-TRAB-COVID19 program is efficient to decrease the risk of infection among the HCWs. Our findings suggest that the implementation of a similar program at a national level can be helpful to provide a safe environment to HCWs and to prevent the collapse of health institutions.

## Introduction

The novel coronavirus disease 2019 (COVID-19) is a current pandemic that emerged in China in late December 2019. The etiologic agent of COVID-19 is the severe acute respiratory syndrome coronavirus 2 (SARS-CoV-2) (1). SARS-CoV-2 infection can pass from a person to another through the inhalation of respiratory droplets from the infected patients, where the virus can stay alive for 3 h (2).

Evidence shows that the use of physical distancing (1 m), optimum use of face masks, respirators, and eye protection is helpful to avoid the person-to-person virus transmission (3). Despite the implementation of such policies, on February 13th, 2021, the World Health Organization (WHO) reported that there were 107,838,255 confirmed cases of COVID-19 along with 2,373,398 deaths worldwide (4). Nationally, the Mexican epidemiologic data reported 1,992,794 confirmed cases of COVID-19, and 174,207 deaths as on February 15th, 2021 (5).

A notable proportion of worldwide reported cases come from the healthcare workers (HCWs). In this regard, science communities have remarked the need for the development of operational guidance in the field of Occupational Hygiene (6). These protocols should describe the recommended management to prevent SARS-CoV-2 spreading in the diverse clinics areas, from those that performe aerosol-generating procedures to gynecological outpatient clinic, since the latter represent potentially affected areas (7, 8).

On February 24th, 2020, China reported that 3,387 (4.4%) of 77,262 total COVID-19 patients were HCWs of which 23 died (9). Whereas, on April 9th, 2020, United States of America (USA) reported 9,282 (16%) patients of COVID-19 identified as HCWs, of which 10% were hospitalized, and 27 died (10).

An Amnesty International report mentioned that Mexico has high a mortality rate of COVID-19 among HCWs, suggesting that Mexico has more than a triple of deaths as compared to USA (11). Agren (12) also mentioned, “…shortages of supplies and tests, along with a lack of hospital infrastructure and even proper training” are factors that promote the high infection rate of SARS-CoV-2 among the HCWs in Mexico. Recently, an analysis of the data collected by the National Epidemiological Surveillance System in Mexico City on July 5th, 2020 reported that 35,095 HCWs were tested for SARS-CoV-2 of which 11,226 (31.9%) were positive and lethality occured in 226 (2.01%) cases (13).

The use of personal protective equipment (PPE) is highly recommended to reduce the nosocomial transmission of viruses, because evidence shows that HCWs are at risk of acquiring respiratory virus infections due to routine care of virus infected patients and thus HCW could further spread the virus (14, 15).

Worldwide, it has been documented that there are deficiencies of PPE components such as masks, face shields, disposable scrubs, and gowns which are caused by high demand disrupting the supply chain. To preserve the use of PPE, a field of research is to implement the protocols to extend the use and reuse of PPE material and disinfection protocols (16–18).

The *Instituto Nacional de Enfermedades Respiratorias Ismael Cosio Villegas* (INER) is a significant and referenced institute dealing with respiratory diseases in Mexico. As a strategy to handle the COVID-19 pandemic, since March 1st, 2020, the Mexican Health Ministry designated the transformation of some health institutions, including INER, to provide care for COVID-19 patients exclusively.

INER implemented the Program of Occupational Medicine for INER workers suspect of SARS-CoV-2 infection (INER-POL-TRAB-COVID19) which was launched on March 1st, 2020. The main objective of INER-POL-TRAB-COVID19 is to establish a protocol to mitigate the risk of SARS-CoV-2 infection and death among INER-HCWs.The methodology included (a) capacitation for the adequate use of PPE, and (b) follow-up of HCWs to analyze the nosocomial incidence of COVID-19. This program required the integration of several departments within the institute for the establishment of adequate practices to maintain the safety of INER-HCWs while dealing with the global limitations of the shortages in the available PPE equipment. Thus, the main aim in this study was to evaluate the efficiency of the INER-POL-TRAB-COVID19 program to mitigate the SARS-CoV-2 infection risk among the INER-HCW.

## Methods

### Ethics Approval

This study was approved by the Ethics committee of the *Instituto Nacional de Enfermedades Respiratorias “Ismael Cosio Villegas”* (protocol code E10-20), and following the principles stipulated in the Helsinki Declaration. All HCWs granted written informed consent to use the collected data for research.

### Institutional Situation

In response to the COVID-19 pandemic, the Mexican Health Ministry released a circular to convert some hospitals to attend to COVID-19 patients exclusively. Currently, INER is the principal national reference insitute to handle COVID-19 patients. It has 250 beds and 200 respiratory ventilators to support patients in critical condition.

To decrease the risk of SARS-CoV-2 infection among the HCWs of the Institute, the departments of Occupational Medicine, Biosecurity, and Teaching have provided daily training courses of the correct use of PPE, starting from the end of January 2020. These courses were designed to be attended in person by HCWs, and virtual resources were also provided in which the training videos were uploaded on the institutional webpage among other platforms like YouTube. Besides, one group of workers was trained to have a “shadow” function of supervising the correct use of PPE before and after the INER-HCWs could enter the exclusive areas to attend to COVID-19 patients.

### Study Populations

The program INER-POL-TRAB-COVID19 has specifically targeted to aim and establish a follow-up of INER-HCWs to analyze the nosocomial incidence of COVID-19. This study reports the evaluation of the INER-POL-TRAB-COVID19 program from March 1st to October 1st, 2020.

INER-HCWs were divided into two groups, (1) frontline workers (FL-W) and (2) non frontline workers (NFL-W). FL-W are all those who attend to patients positive for SARS-CoV-2 infection in the intensive care units. FL-W entail the units of nurses, physicians, stretcher-bearers, imaging technicians, inhalation therapists, and laboratory clinicians. NFL-W are those related to the following units: administration, nutrition, biomedicine, pharmacy, quartermaster, research, laundry, security, syndicate, and social workers.

It is essential to realize that the Mexican Health Ministry has suggested that all those HCWs who suffer from comorbidities such as hypertension, diabetes mellitus, obesity, or older than 60 years of age, were excused to stay in the hospital because they are tagged as a high-risk and vulnerable group of workers. Currently, workers of this group stay at home, but some of them have requested a SARS-CoV-2 test in the INER facilities.

### Procedure Description of INER-POL-TRAB-COVID19 Program

It was made obligatory for INER-HCWs to inform the departmental head whenever they manifested any symptoms related to COVID-19. Afterwards, the subject, along with the direct contacts, were sent to the Occupational Medicine Clinic for clinical revision to identify newly infected and probably asymptomatic patients. Following the clinical revision, demographic and epidemiological advice was provided, and an oro/nasopharyngeal swab was performed for SARS-CoV-2 test by RT-PCR.

**Figure 1:**
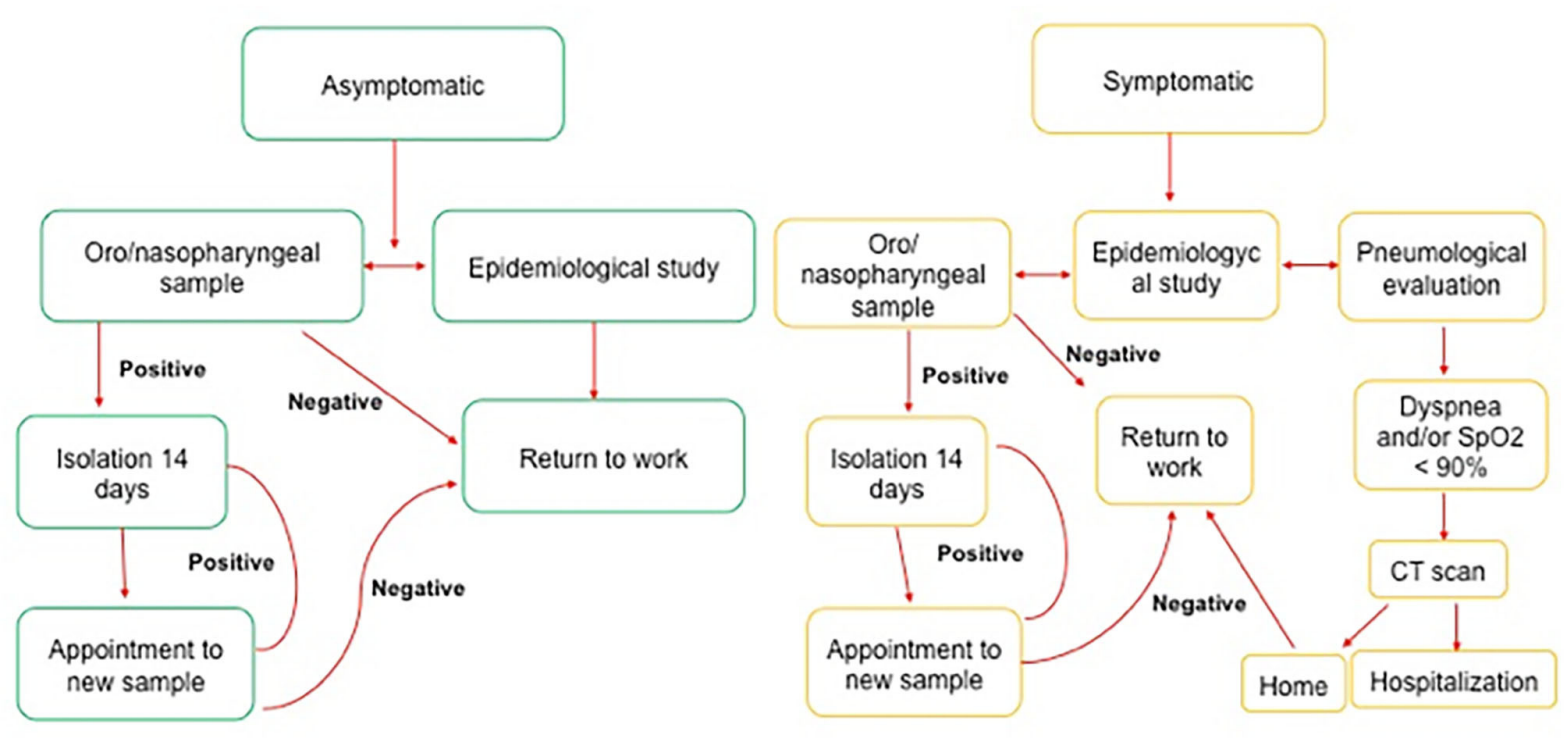
Workflow followed in the INER for the implementation of the INER-POL-TRAB-COVID19 program.

In Figure 1, the follow-up of confirmed asymptomatic or symptomatic patients is shown. Briefly, the INER-HCWs negative for the SARS-CoV-2 test were reincorporated to work, whereas the following steps were considered for the INER-HCWs positive for the SARS-CoV-2 test:

- Clinical checking, including the evaluation of oxygen saturation, fever, dyspnea, anosmia, diarrhea, and fatigue. These among other symptoms, were used to determine if the diagnostic of the INER-HCWs was mild, moderate, or severe illness.
- INER-HCWs with low oxygen saturation and CT scan with suggestive images of pneumonia received a diagnosis as a patient in severe condition and were hospitalized. The Centers for Disease Control and Prevention (CDC) defines severe illness as an individual having (a) a respiratory frequency of >30 breaths per minute, (b) a saturation of oxygen (SpO_2_) <94% (at sea level) but patients with chronic hypoxia must exhibit a decrease from baseline of >3%, (c) a ratio of the arterial partial pressure of oxygen to fraction of inspired oxygen (PaO_2_/FiO_2_) of <300 mmHg, and (d) a lung infiltrate of >50% (19).
- INER-HCWs diagnosed with a mild or moderate condition were sent to home isolation. CDC defines mild illness as an individual who has any of the COVID-19 symptoms without exhibiting any difficulty in breathing, dyspnea, or abnormal chest imaging. Whereas, moderately ill patients are the individuals who have evidence of lower respiratory disease by clinical assessment or imaging and a SpO2 ≥94% (at sea level) (19).
- Another test of SARS-CoV-2 was conducted after 14 days of subject's isolation. If the result of the new test was negative, then the INER-HCW was returned to work. However, if the result was again positive, the INER-HCW was isolated again and placed in the waiting list for another test of SARS-CoV-2 which was repeated every 14 days until obtaining a negative result, which was the only case in which the INER-HCWs could be returned to work.
- HCWs re-incorporated to workplace should use all the safety measures implemented in the Institute, including the use of face shield, face mask, social distance, and a regular extensive hand wash.

### Clinical Probe to Identify SARS-CoV-2 in Throat Swab

For the purpose of the extraction of RNA, around 200 μl of universal viral transport (where the oro/nasopharyngeal swab was deposited) was sampled to extract the genetic material. The extraction process was performed using the QIAamp RNA Viral Mini (Qiagen) kit, and the genetic material was eluted in 140 μl of elution buffer. Oligonucleotides were synthesized and provided by T4-OLIGO. The primer sequences (5′-3′) used to detect target genes for SARS-CoV-2 are summarized in Supplementary 1. These primers were approved by the National Institute of Epidemiological Reference (InDRE).

### Operational Definitions

The following definitions are outlined to improve the context of the problem:

Communitarian contagion: An infection conceived outside of the hospital, this means it was not obtained facing inward or intensive care unit patients. It was classified through the use of a questionnaire (Supplementary 2).

Nosocomial contagion: HCW facing patients in the ward or intensive care unit. It was classified through the use of a questionnaire (Supplementary 2).

Traditional PEE: Masks, face shields, gloves, gowns, protector lens, etc. The use of these items was verified with the questionnaire (Supplementary 2).

Modified PEE: Cotton inner gown instead of a disposable inner gown. Its usage was verified with the questionnaire (Supplementary 2).

### Statistical Analysis

Descriptive statistics were used to summarize the data; results are reported using the mean and standard deviation, categorical variables were summarized as counts and percentages. The analysis was performed with Microsoft Excel 16.16.27 (201012), and Stata 15.1 software (StataCorp).

## Results

### INER-POL-TRAB-COVID19 Program

On March 1st, 2020, through the Clinic of Occupational Medicine, INER implemented the INER-POL-TRAB-COVID19 program which has the aim to provide better care conditions of INER-HCWs and to decrease the nosocomial incidence and mortality by COVID-19. Figure 1 provides a summary of the organization workflow to handle asymptomatic (green) or symptomatic (yellow) INER-HCWs subjects with COVID-19.

### A Third-Quarter Part of the Total INER-HCWs Has Been Evaluated to SARS-CoV-2 Infection Within the INER-POL-TRAB-COVID19 Program

On September 30th, 2020, the INER had a total base of approximately 3,500 HCWs. A minimal variation (±2%) in the total number of HCWs is possible because INER has been employing new personnel since March, considering the excessive work conditions combined with stress and difficulty to work with critical patients. Moreover, new workers were employed for the replacement of the personnel who willingly resigned the contract.

On October 1st, 2020, from a total 3,500 INER-HCWs, 2,505 were tested to SARS-CoV-2 infection (hereafter called INER-HCWs-T) under the INER-POL-TRAB-COVID19 program. This means that 71.5% of the total workers in the Institute were evaluated for SARS-CoV-2 infection.

The baseline characteristics of the INER-HCWs-T group are: mean age of 36 years (min 18, max 68) of which 900 were men (36%), 1,605 were female (64%), 1,937 (77%) were FL-W, and 568 (23%) were NFL-W.

### No Mortality and 3.8% Incidence of SARS-CoV-2 Nosocomial Infection Due to the Implementation of the INER-POL-TRAB-COVID19 Program

Among the 2,505 INER-HCWs-T, 366 (15%) were positive for SARS-CoV-2 infection, which represents 10.4% of the total INER-HCWs. Among these 366 positive cases, 232 (63%) were diagnosed with a communitarian infection, and 134 with nosocomial infection. The latter represents 5% of the INER-HCWs-T population, which also means that only 3.8% of the total INER-HCWs had a SARS-CoV-2 nosocomial infection.

Among these 366 INER-HCWs positive for SARS-CoV-2, 26 (7%) required hospitalization because their clinical situation was diagnosed as severe. However, three INER-HCWs died during this period, two of them belonged to the vulnerable group so the infection was considered as communitarian since the patients had stayed at home. As for the third case, death was caused by acute myocardial injury although this patient was actively working in the hospital. However, it was not sure if this patient had COVID-19 because the subject died in his house.

It is essential to note that among these 366 INER-HCWs positive for SARS-CoV-2 infection, 282 (77%) were FL-W and only 84 (33%) were NFL-W. Our data can confirm that FL-W group is at a higher risk of infection by SARS-CoV-2 (*p* = 0.003). Table 1 shows that nurses have the highest tally of SARS-CoV-2 infection in the FL-W group, whereas administrative personal has the highest tally in NFL-W group.

**Table 1:** Assigment of institute workers positive to SARS-CoV-2 infection.

**Adscription**	**Total number (*n* = 366)**	**Percentage (%)**
Nursing	166	45
Administrative	50	14
Physician	45	12
Quartermaster	26	7
Inhalation therapy/Rehabilitation	15	4
Research	13	4
Nutrition	10	3
Imaging	11	3
Clinical laboratory	8	2
Laundry	4	1
Stretcher-bearer	6	1.6
Maintenance/Transport	6	1.6
Pathology	3	0.9
Social work/Psychology	3	0.9

### INER-HCWs Infected With SARS-CoV-2 Display Predominantly Only One Comorbidity, the Most Frequent Was Obesity

From INER-HCWs positive for SARS-CoV-2, 77 (21%) displayed comorbidities; 55 (71%) had only one comorbidity, 18 (23%) had two comorbidities, and 4 (6%) showed three or more comorbidities. As reported in Table 2, the most frequent was obesity, which was exhibited in 51 (66.2%) INER-HCWs positive for SARS-CoV-2 infection.Obesity was followed by arterial hypertension presented in 15 (19.4%) of these patients.

**Table 2:** Comorbidities showed by institute workers positive to SARS-CoV-2.

**Comorbidity**	**Total number (*n* = 77)**	**Percentage (%)**
Obesity	51	14
Arterial hypertension	15	4
Smoking	10	3
Asthma	10	3
Allergies	7	2
Diabetes mellitus	5	1.3
HIV	1	0.3
Others	8	2

### The Majority of the INER-HCWs Infected With SARS-CoV-2 Required Up to Four RT-PCR Tests to Recover From a Positive Diagnosis, and to Be Negative for SARS-CoV-2 Infection

The follow-up of INER-HCWs positive for SARS-CoV-2 was fundamental to the success in the reduction of SARS-CoV-2 nosocomial infections. As described in the methods section, INER-HCWs positive for SARS-CoV-2 with mild symptoms were isolated in their homes and a new RT-PCR test was performed every 14 days for as many times as necessary. INER-HCWs were allowed back to work once the test result was negative. Table 3 shows the number of RT-PCR test performed on each INER-HCWs positive for SARS-CoV-2. It was observed that 26% of the subjects required the test three times and 27% of the subjects required the test four times. It means that 53% of the INER-HCWs positive for SARS-CoV-2 were reincorporated to their work areas after 42–56 days of the first diagnosis.

**Table 3:** Days from the first SARS-CoV-2 positive diagnosis for institute workers until testing negative.

**Number of tests**	**Total number of patients (366)**	**Percentage (%)**	**Days to negative result**
1	12	3	14
2	64	17	28
3	94	26	42
4	95	27	56
5	41	11	70
6	38	10	84
7	14	4	98
8	7	1.7	112
9	1	0.3	126

### Low Frequency of SARS-CoV-2 Nosocomial Infection Was Not Affected Even With Modifications in the PPE

A shortage of PPE components is not an exclusive problem of Mexico; instead, high demand and a disruption in the supply chain is a worldwide issue (16–18). Thus, it was necessary to modify the PPE which was avalaible on May 11th, 2020, when the dispensable gown was replaced by a cotton gown.

On May 10th, 2020, there were 903 INER-HCWs evaluated for SARS-CoV-2 test infection and 168 (19%) had a positive confirmation of COVID-19. From May 11th to October 1st, 2020, 1,602 INER-HCWs were evaluated for SARS-CoV-2 infection. Among these 1,602 INER-HCWs, 199 (12%) had a positive confirmation of COVID-19, this decrease in the percentage of INER-HCWs positive for SARS-CoV-2 was statistically significant (*p* = 0.001). Thus, the change between dispensable and cotton gowns did not increase the incidence SARS-CoV-2 infection within INER-HCWs, in fact, the incidence decreased.

## Discussion

INER is the principal reference institute of respiratory diseases in Mexico, and it led the attention of patients during the H1/N1 flu pandemic in 2009 and the learnt experiences had contributed toward the skill improvement for better management (20).

Avoiding the collapse of any health institute is fundamental mainly during a challenging time like a global pandemic, which depends a lot on the health and safety of the HCWs. Starting from this principle, on March 1st, 2020 the INER implemented policies focusing on the health care of INER-HCWs.

INER organization started its operation at the end of January 2020. INER-POL-TRAB-COVID19 program organized various departments to provide efficient training to all workers regarding the use of PPE and biosecurity rules. Thus, when the first COVID-19 patient was received on February 28th, 2020, the institutional organization was already implemented.

Our data have demonstrated that the implementation of this Occupational Medicine program in our Institute has been successful in maintaining a low frequency of incidence and mortality by SARS-CoV-2 among the INER-HCWs. We are reporting that INER has an incidence of 3.8% of SARS-CoV-2 nosocomial infection and null mortality.

Earlier reports have indicated that Mexico is a leading country in the death toll among HCWs; however, those reports are insufficient to reveal if the deaths were related to nosocomial or communitarian infection (11, 12). That point is of greater importance because only in this way, it is possible to identify if the high incidence and deaths by SARS-CoV-2 in HCWs are caused by health system deficiencies as referred by Agren (12). Moreover, this author also reported that “…1,410 healthcare workers had died, almost half of them were doctors” (12). In this matter, our data is consistent in that FL-W has high possibility to be infected, however, our analysis showed that nursing staff have the highest infection rate compared to physician and inhalation therapy staff.

Antonio-Villa et al. (13) reported an analysis of data collected by the National Epidemiological Surveillance System in Mexico City. Their results have shown that the mean infection rate of SARS-CoV-2 in HCWs of Mexico City is 31.9%. Nevertheless, it was not possible to differentiate between nosocomial and communitarian infection. Thus, the implementation of the INER-POL-TRAB-COVID19 program has reduced the incidence of SARS-CoV-2 infected HCWs by 10.4%, independent to the origin (nosocomial or communitarian) of contagion. It means that the program is efficient to maintain the health of the personnel. Moreover, the decrease in the infection cases when the dispensable gown was replaced by a cotton gown, may have been because the INER-HCWs were more careful when handling PPE.

The authors of this work have provided evidence that the establishment of the INER-POL-TRAB-COVID19 program has been highly efficient to maintain a low frequency of infection and mortality by SARS-CoV-2 in HCWs, for the sake of one of the most prominent health institutes in Mexico. Thus, from the author's perspective, it is an efficient option to decrease the high prevalence of SARS-CoV-2 infection in HCWs in Mexico. Therefore, the Mexican Health Ministry must consider implementing a similar program in the “health centers exclusively dedicated to COVID-19 patients” to provide a safe and healthy environment for the HCWs, which can aid to prevent any health institute from collapsing.

## Data Availability Statement

The original contributions presented in the study are included in the article/Supplementary Material, further inquiries can be directed to the corresponding author/s.

## Ethics Statement

The studies involving human participants were reviewed and approved by Ethic Committee of Instituto Nacional de Enfermedades Respiratorias. The patients/participants provided their written informed consent to participate in this study.

## Author Contributions

MS, LC-G, AC-M, and IB-R searched the literature. MS, LC-G, AC-M, and IB-R conceived and designed the study. MS, LC-G, AC-M, PS-D, JR-P, JS-H, and IB-R examined and interpreted the experimental data. MS, AC-M, MM-A, ND-V, AV-M, JM-O, EB-V, FS-G, HP-G, DA-M, EL-S, FV, LV-G, and SP-P established the follow-up of the workers and analyzed clinical data. MS, AC-M, PS-D, JR-P, and JS-H designed the implementation of the INER-POL-TRAB-COVID19 program. MS, LC-G, and IB-R contributed to the manuscript preparation. LC-G wrote the manuscript with input from all co-authors. All authors contributed to the article and approved the submitted version.

## Conflict of Interest

The authors declare that the research was conducted in the absence of any commercial or financial relationships that could be construed as a potential conflict of interest.
