# Supplementary material for: Low Incidence and Mortality by SARS-CoV-2 Infection Among Healthcare Workers in a Health National Center in Mexico: Successful Establishment of an Occupational Medicine Program
Source: Front Public Health. 2021 Apr 13;9:651144. doi: 10.3389/fpubh.2021.651144 (PMC8076634; doi:10.3389/fpubh.2021.651144)
Supplement: Supplementary file 1 [file Table_1.pdf]

Title: Low incidence and mortality by SARS-CoV-2 infection among healthcare workers in a Health National Center in Mexico: successful establishment of an occupational medicine program

Supplementary material

Table 1. Primer sequences (5'-3') approved by InDRE and utilized to detect target genes for SARS-CoV-2

**Primer/Probe name**	**Direction**	**Target**	**Sequence (5'- 3')**
E_Sarbeco_F1	Primer Forward		ACAGGTACGTTAATAGTTAATAGCGT
E_Sarbeco_R2	Primer Reverse	SARS-CoV-2 *E* gene	ATATTGCAGCAGTACGCACACA
E_Sarbeco_P1	Probe		FAM-ACACTAGCCATCCTTACTGCGCTTCG-BHQ
RdRP_SARSr-R1	Primer Forward		CARATGTTAAASACACTATTAGCATA
RdRP_SARSr-F2	Primer Reverse	SARS-CoV-2 *RdRp* gene	GTGARATGGTCATGTGTGGCGG
MAPGD_19_RdRP_SARSr	Probe		CAGGT(G)(G)AACC[BHQ1-Dt]CATCA(G)(G)AGATGC
Rnase P for	Primer Forward		AGATTTGGACCTGCGAGCG
Rnase P Rev	Primer Reverse	Human *RNasa P* gene	GAGCGGCTGTCTCCACAAGT
Rnase P	Probe		FAM-TTCTGACCTGAAGGCTCTGCGCG-BHQ1

Supplementary 1. Primer sequences to identify SARS-CoV-2.
