# Supplementary material for: Low Incidence and Mortality by SARS-CoV-2 Infection Among Healthcare Workers in a Health National Center in Mexico: Successful Establishment of an Occupational Medicine Program
Source: Front Public Health. 2021 Apr 13;9:651144. doi: 10.3389/fpubh.2021.651144 (PMC8076634; doi:10.3389/fpubh.2021.651144)
Supplement: Supplementary file 2 [file Table_2.pdf]

Title: Low incidence and mortality by SARS-CoV-2 infection among healthcare workers in a Health National Center in Mexico: successful establishment of an occupational medicine program

Supplementary material

The questionnaire used to Program of Occupational Medicine (INER-POL-TRAB-COVID19)

1. Name, given and last.
2. Gender.
3. Date of birth
4. Where is your current assignment in the hospital?
5. How much time have you worked in this institute?
6. Why were you sending to SRAS-CoV-2 test?
7. Did you were in a hospital service where there are aerosols?
8. Did you wear your Personal Protection Equipment (PPE)?
9. Did you were in a place with COVID-19 patients?
10. Do you attend directly COVID-19 patients?
11. Did you make aerosol-generating procedures?
12. If the previous answer is yes, What kind of aerosol?
13. Do you wear n95 mask?
14. Do you wear gloves in the attention of patients?
15. Do you wear ocular protection?
16. Did you have some accident with your PPE?
17. What kind of transport do you use to get to the hospital? (Public, private, bike, walking)?
18. In your home, there are people with COVID-19 symptoms?
19. In the last 15 days, Did you stay at a bar/restaurant?
20. Did you stay with more than ten people in a room?
21. Are you working in second place?
22. Are you staying in your home because you are of the vulnerable group?

Supplementary 2. Questionnaire to obtain epidemiological information.
